# Can expelled cells/debris from a developing embryo be used for PGT?

**DOI:** 10.1186/s13048-021-00853-6

**Published:** 2021-08-11

**Authors:** Adva Aizer, Noa Harel-Inbar, Hagit Shani, Raoul Orvieto

**Affiliations:** 1 Department of Obstetrics and Gynecology, Sheba Medical Center, Ramat-Gan, Israel; 2 Sackler School of Medicine, Tel Aviv University, Tel Aviv, Israel; 3 Danek Gertner Institute of Human Genetics, Sheba Medical Center, Ramat-Gan, Israel; 4 The Tarnesby-Tarnowski Chair for Family Planning and Fertility Regulation, Sackler Faculty of Medicine, Tel-Aviv University, Tel Aviv, Israel

**Keywords:** PGT, PCR, Cleavage-stage, Blastocyst, Cell debris, Self-correction

## Abstract

**Background:**

Preimplantation genetic testing (PGT) is offered to a wide range of structural and numerical chromosomal imbalances, with PGT- polymerase chain reaction (PCR), as the method of choice for amplifying the small DNA content achieved from the blastomere biopsy or trophectoderm (TE) biopsy, that might have a detrimental impact on embryonic implantation potential. Since human embryos cultured until Day-5–6 were noticed to expel cell debris/ fragments within the zona pellucida, we aimed to examine whether these cell debris/ fragments might be used for PGT, as an alternative to embryo biopsy.

**Methods:**

Blastocysts, which their Day-3 blastomere biopsy revealed an affected embryo with single-gene defect, and following hatching leaved cell debris/fragments within the zona pellucida were analyzed. Each blastocyst and its corresponding cell debris/fragments were separated and underwent the same molecular analysis, based on multiplex PCR programs designed for haplotyping using informative microsatellites markers. The main outcome measure was the intra-embryo congruity of Day-3 blastomere biopsy and its corresponding blastocyst and cell debris/fragments.

**Results:**

Fourteen affected embryos from 9 women were included. Only 8/14 (57.2%) of embryos demonstrated congruent molecular genetic results between Day-3 embryo and its corresponding blastocyst and cell debris/fragments. In additional 6/14 (42.8%) embryos, molecular results of the Day-3 embryos and their corresponding blastocysts were congruent, while the cell debris/fragments yielded no molecular diagnoses (incomplete diagnoses).

**Conclusions:**

It might be therefore concluded, that in PGT cycles, examining the cell debris/fragments on Day-4, instead of Day-3 blastomere or Day-5 TE biopsies, is feasible and might avoid embryo biopsy with its consequent detrimental effect on embryos’ implantation potential. Whenever the latter results in incomplete diagnosis, TE biopsy should be carried out on Day-5 for final genetic results. Further large well-designed studies are required to validate the aforementioned PGT platform.

## Introduction

Preimplantation genetic testing (PGT) enables the birth of healthy offspring in couples at risk for transmitting a serious genetic disease. PGT is offered to a wide range of structural and numerical chromosomal imbalances, monogenic disease, HLA typing, etc. [1, 2], with PGT- polymerase chain reaction (PCR), as the method of choice for amplifying the small DNA content achieved from the blastomere biopsy.

De Vos et al. have demonstrated that 8-cell embryos having lost two blastomeres by embryo biopsy have a 40% lower implantation potential compared with 8-cell embryos having lost only one blastomere [3]. Moreover, following a 2013 study [4], demonstrating that cleavage-stage biopsy markedly reduced embryonic implantation potential compared to trophectoderm (TE) biopsy, a shift toward blastocyst rather than cleavage-stage biopsies appeared, mostly in PGT-Aneuploidy (PGT-A) setups. Of notice, a follow-up study by the same group [5] showed that higher DNA content in the TE biopsies is associated with lower live birth, emphasizing that TE biopsies might also have a detrimental impact on embryonic implantation potential. Moreover, a negative impact on implantation was also demonstrated if the biopsied blastocyst is not expanded and hatching at the time of biopsy [6]. Of interest, even the recently published STAR study has speculated that PGT-A did not improve implantation or OPR per embryo transfer in the younger patients, possibly because of the detrimental effect of the biopsy previtrification on the embryo viability [7].

**Fig. 1:**
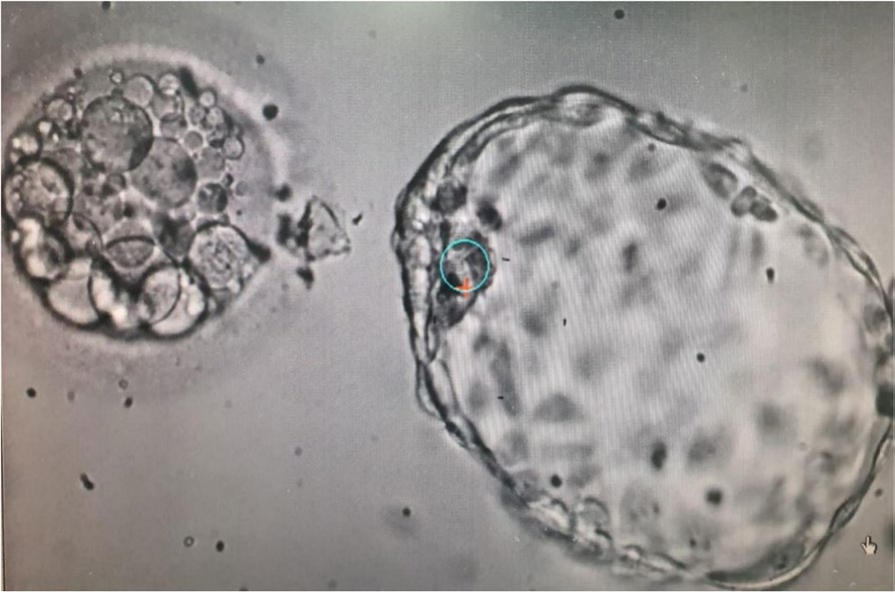
Day 5 hatched blastocyst and its original zona pellucida containing leftovers of cell debris

In our PGT program, DNA extraction is obtained during the cleavage stage, where one blastomere from Day 3 embryo is extracted and undergo genetic diagnosis. In our routine clinical practice, following Day-3 biopsy, healthy embryos are transferred on Day-4 or 5, and the affected embryos are discarded. When these affected embryos were cultured until Day-5–6, we noticed that some of the blastocysts expel cell debris/ fragments within the zona pellucida (Fig. 1). Whole genome amplification of each blastocyst and its corresponding debris have demonstrated that most of blastocysts expelled cell debris with abnormal chromosomal rearrangements [8].

Prompted by the aforementioned observations we sought to examine whether these cell debris/ fragments might be used for PGT, as an alternative to embryo biopsy. Thus, avoiding the embryo injury and the consequent detrimental effect on its implantation potential.

## Patients and methods

Blastocysts, in which their Day-3 blastomere biopsy revealed an affected embryo with single-gene defect, were donated by couples undergoing PGT treatment at the Sheba Medical Center. Only blastocysts cultured in closed system using the time-lapse EmbryoScope™ incubator, that following hatching leaved cell debris/fragments within the zona pellucida were analyzed. Each blastocyst and its corresponding cell debris/fragments were separated and underwent the same genetic analysis, as their originator Day-3 embryo.

PGT was based on multiplex PCR programs designed for haplotyping using informative microsatellites markers. The molecular diagnosis and the laboratory procedures were thoroughly presented elsewhere [9]. The operator of the molecular analysis (N.H.I) was blinded to the samples’ sources.

Molecular diagnoses of each sample were classified as follows [9]: Complete diagnosis – unaffected or affected embryo according to the genetic disorder examined; Incomplete diagnosis—suspected allele dropout or recombination; PCR failure – no DNA is available for diagnosis; and Abnormal – the embryo has abnormal assembly of alleles – i.e. any structure different from one maternal and one paternal alleles matching the known haplotype, e.g. trisomy, monosomy or uniparental disomy.

Patients’ characteristics, their genetic disease and the embryological and PGT variables were retrieved from the patients’ medical charts. The molecular diagnosis of each embryo was correlated to its corresponding blastocyst and cell debris/fragments.

The study required no modification of patient’s routine follow-up or treatment. Informed consent was obtained from all patients before participation in the study, and the study was approved by our Institutional Clinical Research Committee (IRB SMC-19-6140).

## Results

Fifteen affected embryos from 9 women (age 34.0 ± 6.0 yrs) that were cultured in closed system using the time-lapse photography (EmbryoScope™ incubator) in our PGT program and expelled cell debris/ fragments outside the intact embryo, were evaluated. All Day-3 embryos were of top-quality; i.e. 7–9 equal blastomeres with no fragmentations.

Patients’ genetic abnormalities, the embryological and PGT results obtained from the Day-3 embryos and their corresponding blastocysts and cell debris/ fragments are presented in Table 1. While 2 embryos expelled 2–8 cells, 2 embryos expelled only cell debris. Molecular genetic results from both expelled cells or debris resulted in either complete diagnosis, PCR failure, incomplete or abnormal diagnoses.

**Table 1:** Patients characteristics and their embryological and PGT analyses

**Patient**	**Age**	**Disease/Etiology**	**Embryo #**	**Day 3 quality**	**Day 3 Diagnosis**		**Day 5/6 quality**	**Day 5/6 Diagnosis**
(A)								
A	33	2p 16.3 microdeletion	1	7A	Affected	**Embryo**	3BB	Affected
						**Debris**	2 cells	Affected
			2	8A	Affected	**Embryo**	6AA	Affected
						**Debris**	4 cells	Affected
B	40	Marfan Syndrome	3	8A	Affected & Aneuploidy	**Embryo**	4AB	Affected & Aneuploidy
						**Debris**	3 cells	Affected
C	43	Ataxia Telengiectasia	4	9A	Unaffected & Aneuploidy	**Embryo**	4BA	Unaffected & Aneuploidy
						**Debris**	3 cells	Unaffected & Aneuploidy
D	29	Creutzfeld Jakob Disease	5	8A	Affected	**Embryo**	6AA	Affected
						**Debris**	5 cells	Affected
E	34	Machado Joseph Disease	6	7A	Affected	**Embryo**	G2	Affected
						**Debris**	Cell fragments	Affected & Aneuploidy
F	37	Neurofibromatosis type I	7	8A	Affected	**Embryo**	6BB	Affected
						**Debris**	4 cells	Affected & Aneuploidy
			8	8A	Affected	**Embryo**	6AB	Affected
						**Debris**	3 cells	Affected & Aneuploidy
(B)								
B	40	Marfan Syndrome	9		Affected & Aneuploidy	**Embryo**	6BB	Affected
						**Debris**	8 cells	Allele drop out
G	32	Synopolydactyly II	10		Affected	**Embryo**	6AA	Affected
						**Debris**	Cell fragments	Allele drop out
H	33	Huntington’s disease	11		Affected	**Embryo**	6AA	Affected
						**Debris**	2 cells	Foreign DNA & Aneuploidy
I	24	Nager Syndrome	12		Affected	**Embryo**	4BC	Affected
						**Debris**	2 cells	Undetectable
			13		Affected	**Embryo**	6BA	Affected
						**Debris**	2 cells	Allele drop out
			14		Affected	**Embryo**	5BB	Affected
						**Debris**	3 cells	Unsatisfactory diagnosis

Only 8/14 (57.2%) of embryos demonstrated congruent molecular genetic results (complete diagnoses) between Day-3 embryo and its corresponding blastocyst and cell debris/fragments. In four of which, either the blastocyst or the cell debris/fragment demonstrated additional abnormal karyotype (aneuploidy) (Table 1).

In the remaining 6/14 (42.8%) embryos, molecular results of the Day-3 embryos and their corresponding blastocysts were congruent (complete diagnoses), while the cell debris/fragment yielded no conclusive molecular diagnoses, either because of PCR failure, incomplete or abnormal diagnoses (Table 1).

## Discussion

In the present study of patients undergoing IVF treatment cycle, utilizing PGT based on multiplex PCR programs, the molecular diagnosis of all Day-3 embryos were congruent to their corresponding blastocysts, while in only 57.2% of embryos, the molecular diagnosis of the cell debris/fragment were congruent to the initial Day-3 genetic diagnosis.

According to the ESHRE PGD Consortium data collection XIV-XV [10] on PGT cycles for monogenic diseases, day 3 cleavage-stage embryo biopsy was still the most frequently used (93% of cycles), while the use of blastocyst biopsy remained low (2%). Moreover, PCR was the most widely used first-line method of DNA amplification (93% of cycles). Results that were very similar to the previous ESHRE PGD Consortium data collection XIII. The present study further validates and strengthens the accuracy of Day-3 blastomere biopsy, as compared to Day-5 TE biopsy for PGT.

In the present study, we could demonstrate that complete molecular diagnosis of cell debris/fragment expelled from the embryos might be used for PGT, avoiding embryo biopsy. Of notice, whenever the molecular diagnoses of the cell debris/fragments revealed complete diagnoses, i.e. unaffected or affected embryos according to the genetic disorder examined, the results were in accordance with the Day-3 and Day-5 TE biopsies. However, when the molecular diagnoses were incomplete (suspected allele dropout or recombination), PCR failure (no DNA is available for diagnosis), or abnormal (the embryo has abnormal assembly of alleles – i.e. any structure different from one maternal and one paternal alleles matching the known haplotype, e.g. trisomy, monosomy or uniparental disomy), we cannot rely on the cell debris/fragments, and a different genetic method should be implemented. Of notice, in 57.2% of the embryos, we could rely on the molecular diagnosis of expelled cell debris/fragment and avoid embryo biopsy, with its possible detrimental effect on embryos development. However, in 42.8% of the embryos, TE biopsy and repeated molecular diagnosis are required. Interventions that increase the cost, unless the cell debris/fragments biopsy will improve/optimized. We believe that further large studies will enlighten the cost-effectiveness implication of the molecular diagnosis of cell debris/fragment expelled from the embryos for PGT.

We also observed that in 3 of the embryos (#6,7 and 8), the cell debris showed that not only the embryo was affected, as demonstrated by the embryo biopsy utilizing PGT based on multiplex PCR, but also characterized by aneuploidy. This observation is in accordance with our previous study, demonstrating that human embryos have the ability of self- correction [8]. We showed that human embryos not only eliminate/expel complete abnormal blastomeres, but also chromosomal-abnormal cell debris/fragments. Whole genome amplification of each blastocyst and its corresponding debris demonstrated that 63.6% of blastocysts expelled cell debris with abnormal chromosomal rearrangements. Moreover, among the 9 euploid blastocysts, 5 (55.5%) had expelled aneuploid debris [8].

Prerequisite to Day-5 TE biopsy is laser hatching, i.e. opening of the zona pellucida on day 3 of in vitro development, enabling the TE cells herniation through that opening and their biopsy on day 5. We might therefore suggest deferring the zona pellucida opening to Day-4 [11], enabling the retrieval of the cell debris/fragments for the molecular diagnosis. If the results of latter molecular diagnosis, on the following day (Day-5), will be complete/conclusive, the embryo is diagnosed and can be handled accordingly. Of notice, while comparing the efficacy and clinical outcome of PGT-M undertaken on Day-3 or Day-4 embryos, Day-4 embryo biopsy was found to be feasible and yielded comparable and even higher ongoing pregnancy rate if undertaken at the morula stage [11].

However, if the results of the molecular diagnosis of the cell debris/fragments are inconclusive, as shown in our study, due to PCR failure, incomplete or abnormal diagnoses; or the cell debris showed unaffected embryo, as demonstrated by the embryo biopsy utilizing PGT based on multiplex PCR, but the embryo was aneuploidy, in these cases, the blastocyst that already underwent zona pellucida opening on Day-4, will undergo additional TE biopsy, and will be cryopreserved until achieving the final definite molecular results.

The limitations of our study is the small sample size of affected embryos donated for the study, which are strongly influenced by the ethical concerns limiting human embryos research. If overcome, we believe that larger studies should be undertaken to examine the congruity between both the cell debris/fragments on Day-4 following zona pellucida opening and Day-5 TE biopsies (in all embryos). Moreover, if our observation will be validated, further studies will be needed to verify whether avoiding embryo biopsy by examining the cell debris/fragment will improve embryos implantation potential, as compared to Day-3 blastomere or Day-5 TE biopsies.

Of notice, since these cell debris/fragments are the primary source of cell-free DNA (cfDNA) in the embryo culture media [8], the recently adopted non-invasive PGT-A [10], as another routine adjunct to IVF, should also be re-evaluated carefully. A recent study applying whole genome amplification of human embryos and their cell debris/fragments has demonstrated that 63.6% of blastocysts expelled cell debris with abnormal chromosomal rearrangements [8]. Moreover, 55.5% of ***euploid*** blastocysts expel ***aneuploid*** debris, suggesting a substantial ability to self-correct downstream from the blastocyst stage. Therefore, any chromosomal diagnosis at the blastocyst stage should be considered potentially useless, and this, unfortunately must also include non-invasive PGT-A based on cell-free DNA in spent medium. The high rates of false-positive diagnoses will often lead to disposal of embryos with entirely normal implantation/pregnancy potential. Moreover, human embryos ability for self-correction mechanisms [8, 12–14], together with known chromosomal mosaicism, a common feature of early human embryos development [15], reinforce the crucial need for prenatal diagnosis (by either chorionic villi sampling or amniocentesis) in PGT pregnancies, to validate the molecular PGT results of the growing embryo.

## Conclusion

As mentioned above, further large well-designed studies are required to validate the aforementioned PGT platform. An interim conclusion might be that in PGT cycles, examining the cell debris/fragments instead of Day-3 blastomere or Day-5 TE, is feasible and might avoid embryo biopsy with its consequent detrimental effect on embryos’ implantation potential. Moreover, whenever, no complete diagnosis could be achieved from the molecular diagnosis of the cell debris/fragments, Day-5 TE biopsy should be done, and the blastocyst should be cryopreserved until final genetic results (as routinely practiced).

## Data Availability

All relevant data are within the manuscript.
